# Landscape analysis of matrix metalloproteinases reveals key prognostic markers for prostate cancer

**DOI:** 10.3389/fimmu.2025.1582992

**Published:** 2025-06-18

**Authors:** Wei Li, Xi Wei, Ying Yu, Yuan Tian, Qi Yu, Jun Qiao, Yuewei Tao, Yanfeng Li, Tao Li

**Affiliations:** ^1^ Department of Urology, the Affiliated Hospital of Guizhou Medical University, Guiyang, China; ^2^ Zhejiang Provincial People’s Hospital Bijie Hospital, Bijie, China; ^3^ School of Medicine, University of Dundee, Ninewells Hospital, Dundee, United Kingdom

**Keywords:** prostate cancer, matrix metalloproteinases, immune microenvironment, fibroblasts, MMP11

## Abstract

**Background:**

Prostate cancer (PCa) is the most common male malignancy and significantly impairs patient’s survival. Matrix metalloproteinases (MMPs) play a crucial role in tumor progression, yet the comprehensive role of MMPs in PCa remains unclear.

**Method:**

Data from UCSC and GEO databases were firstly analyzed to evaluate expression characteristics, prognostic value, immune-cell infiltration, tumor-mutation burden (TMB), microsatellite-instability (MSI), immunotherapy sensitivity, and drug sensitivity in PCa. COX-regression analysis was utilized to identify MMPs that affected (Disease-free survival) DFS. Various cellular functional experiments and conditional medium cultivation system were utilized to verify the effect of MMP11 on PCa cells. Subsequently, single-cell transcriptome and spatial-transcriptome data was analyzed to explore the regulatory effect of MMP11 on microenvironment.

**Result:**

Most MMPs exhibit differential expression between tumor and normal tissues, with specific MMPs correlating with pathological features of PCa. Among 24 MMPs analyzed, MMP11 was uniquely associated with shorter DFS. High MMP11 expression correlated with increased infiltration of regulatory Tregs and M2 macrophages, elevated immune checkpoint molecule expression, higher TMB, MSI, and enhanced immunotherapy sensitivity. MMP11 suppression inhibited PCa cell proliferation, migration, invasion, and epithelial-mesenchymal transition.MMP11 was predominantly expressed in fibroblasts and linked to the establishment of an immunosuppressive tumor microenvironment. Targeting MMP11 in cancer-associated fibroblasts reversed their pro-tumorigenic effects on PCa progression. Finally, MMP11 is broadly upregulated across malignancies and associated with poor prognosis in multiple cancer types.

**Conclusion:**

This study comprehensively explored the role of MMPs in PCa. Noteworthy, we further proved that MMP11 significantly promoted PCa probably through reprogramming of tumor microenvironment, which might provide a promising-target for PCa treatment.

## Introduction

Prostate cancer (PCa) is the most common male malignancy and causes the second most cancer-related deaths, with approximately 1.4 million new cases and 396,000 deaths occures worldwide in 2024, posing serious threats to men’s health and leading tremendous healthcare burden (1). Although new therapeutic approaches including the novel hormone therapy, immunotherapy, and homologous recombination inhibitors has been explored, individuals with advanced or metastatic disease still experience poor survival, especially progressing to castration-resistant prostate cancer stage (1–3). Thus, it is essential to explore reliable molecular markers and therapeutic targets for PCa patients.

As a zinc-dependent family of endopeptidases, matrix metalloproteinases (MMPs) are typically categorized into six groups based on their functional and structural characteristics, including collagenases, gelatinases, stromelysins, matrilysins, MMPs activated by furin protease, and other secreted MMPs (4–9). These enzymes are intricately involved in the remodeling and degradation of extracellular matrix (ECM) proteins to exert regulatory functions during various biological processes like apoptosis, immune tolerance, cell migration, and angiogenesis. Notably, MMPs are closely associated with tumor invasion, metastasis, and progression, while they are also considered as promising biomarkers or therapeutic targets in cancer (9–11). Nevertheless, the precise role of MMPs in PCa remains incompletely understood and warrants further investigation.

In this study, we explored the expression characteristics and prognostic value of 24 MMPs in PCa. Meanwhile, we investigated MMP11 on PCa from the angle of immune-cell infiltration and regulation, tumor mutational burden (TMB), microsatellite instability (MSI), immunotherapeutic sensitivity, and drug sensitivity. Moreover, we investigated the effects of MMP11 on the biological behaviors of PCa cells by various cellular function assays and analyzed its regulatory effects on the PCa microenvironment based on single-cell transcriptome and spatial transcriptome data. Finally, the expression pattern and prognostic value of MMP11 in tumors were explored based on pan-cancer data.

## Materials and method

### Data source and processing

Transcriptomic and clinical data spanning 34 cancer types and 31 normal human tissues were obtained from The Cancer Genome Atlas (TCGA) and Genotype-Tissue Expression (GTEx) projects via the UCSC Xena platform (https://xena.ucsc.edu/). Somatic mutation data for TCGA prostate adenocarcinoma (TCGA-PRAD) were also retrieved from this platform. Single-cell RNA-seq data (GSE185344) and bulk RNA-seq datasets (GSE21032, GSE70768, GSE70769, GSE116918) with matched clinical metadata were downloaded from the Gene Expression Omnibus (GEO). Spatial transcriptomic data from one prostate cancer patient were acquired through the 10x Genomics platform (https://www.10xgenomics.com/). The combined GEO cohort (GSE21032, GSE70768, GSE70769) underwent batch effect correction using the R package ‘sva’ with the ComBat algorithm. For GSE185344, samples with extreme cell counts (top/bottom 5% by total UMI) were excluded to mitigate technical variability. Patients lacking biochemical recurrence (BCR) status or follow-up < 1 months were removed from TCGA-PRAD and all GEO cohorts to ensure clinical relevance.

### Pathway enrichment analysis and genomic mutation analysis

Single-sample gene set enrichment analysis (ssGSEA) was performed using the GSVA R package (v1.46.0) with the Hallmark gene set (v7.5.1, MSigDB). Pathway activity scores were calculated for each TCGA-PRAD patient, and differential pathway activation between MMP11-high and MMP11-low groups was assessed via the limma R package (v3.56.2). The maftools R package (v2.16.0) was employed to process somatic mutation data, including variant annotation, tumor mutation burden (TMB) calculation, and identification of significantly mutated genes (SMGs) using MutSig2CV.

### Immune microenvironment analysis and immune response prediction

Immune cell abundance was estimated using the CIBERSORT algorithm (12), while the R package estimate (13) was employed to compute stromal/immune scores and tumor purity, reflecting the tumor microenvironment composition. To predict immunotherapy responsiveness, the immunotherapy response score was calculated via the EaSIeR package (14), which integrates transcriptomic signatures of immune checkpoint blockade (ICB) sensitivity. To validate our findings, we assessed the impact of MMP11 expression on ICB efficacy using the IMPACT platform (http://www.brimpact.cn) in two independent anti-PD-1/PD-L1-treated cohorts: melanoma (Liu_2019) and clear cell renal carcinoma (Chekamte_010).

### scRNA processing

To ensure data reliability, we implemented stringent quality control filters: (1) Cells with <200 detected genes (low-information cells) or >3,000 genes (potential doublets) were excluded. (2) Cells with total UMI counts ≤200 were removed to ensure adequate sequencing depth. (3) Cells exhibiting >10% mitochondrial gene expression (indicative of cellular stress or apoptosis) were discarded.Post-QC, the following analytical workflow was applied: (1) Data were normalized using ‘SCTransform’ to stabilize variance and remove technical noise. (2) Cross-sample batch effects were mitigated via ‘Harmony’ integration. (3) Feature scaling (ScaleData) was performed to equalize gene expression variances. (4) Principal Component Analysis (RunPCA) was conducted to extract the top 50 principal components. (5) Uniform Manifold Approximation and Projection (UMAP) was employed for 2D visualization with optimized parameters: n.neighbors=25, min.dist=0.5, spread=1.0.

### Identification of fibroblast characteristics

To identify marker genes distinguishing fibroblast subpopulations, we performed differential gene expression analysis using the FindAllMarkers function in Seurat (v4.3.0) with the following parameters. For distinct subgroups within the identical fibroblasts cluster, namely the MMP11-positive and MMP11-negative groups, employ the ‘DESeq2’ to assess the DEGs in each subgroup. Subsequently, the top 30 genes exhibiting the highest average expression specificity across fibroblast subtypes were designated as signature genes.

### Cell communication analysis

The R package ‘CellChat’ is utilized to infer the interactions between different cell types. Receptor ligand pairs expressed in at least 10 cells are included in the analysis.

### Spatial transcriptome analysis

ST data were analyzed with Seurat package using similar method as scRNA-seq data.

### Drug sensitivity analysis

To assess therapeutic vulnerabilities, we employed the ‘oncoPredict’ R package (v0.2) to computationally predict the half-maximal inhibitory concentration (IC50) values of 198 FDA-approved or clinically investigated oncology drugs for all patients in the TCGA-PRAD cohort. This pharmacogenomic approach leverages tumor transcriptomic profiles to model drug sensitivity patterns, with IC50 serving as a key pharmacodynamic indicator of intrinsic drug resistance.

### Cell culture and transfection

The human prostate cell lines—normal epithelial cells (RWPE-1) and adenocarcinoma cells (PC3, DU145, and 22RV1)—were obtained from the Cell Bank of the Chinese Academy of Sciences. Human prostatic fibroblasts (HPF) were purchased from Procell Life Science & Technology Co., Ltd. (Wuhan, China). All cell lines were cultured according to the manufacturer’s protocols and maintained at 37°C in a humidified atmosphere containing 5% CO_2_. For MMP11 downregulation in DU145 and HPF cells, transfection was performed using Lipofectamine 8000 (Beyotime Biotechnology, China) and Opti-MEM (Gibco, USA), following the manufacturer’s instructions. Synthetic siRNA constructs—si-NC (negative control), si-MMP11#1, and si-MMP11#2—were synthesized by Sangon Biotech (Shanghai, China). Detailed siRNA sequences are provided in **Supplementary Table S1**.

### Preparation of conditioned medium

To prepare DU145-conditioned medium (CM), DU145 cells were cultured until reaching ~80% confluency. The medium was then replaced with serum-free RPMI 1640 and incubated for 48 hours to collect the CM. The CM was centrifuged at 1500 × g for 10 minutes, filtered through a 0.22 μm membrane, and stored at −80°C until use. HPFs at 50% confluency were treated with DU145-derived CM supplemented with 10% FBS for 72 hours to induce cancer-associated fibroblasts (CAFs) for subsequent analyses. HPF- conditioned medium and CAF-conditioned medium were subsequently harvested from HPFs and transfected CAFs using the same protocol as described for DU145 cells.

### Quantitative reverse transcriptase detection

Total RNA was isolated from cells using TRIzol reagent (Invitrogen) following the manufacturer’s protocol. RNA purity and concentration were quantified via a NanoDrop 2000 spectrophotometer (Thermo Fisher Scientific, USA), with samples retained only if the A260/A280 ratio exceeded 1.8. Reverse transcription to cDNA was performed using the PrimeScript™ RT Reagent Kit (Takara Bio, Japan). mRNA expression levels were analyzed by qPCR with the Premix Ex Taq™ kit (Takara Bio) on a QuantStudio 5 system (Applied Biosystems). All reactions were run in triplicate under the following cycling conditions: 95°C for 30 sec, 40 cycles of 95°C for 5 sec, and 60°C for 34 sec. Primer sequences are listed in **Supplementary Table S2**.

### Western blot detection

Total protein was extracted using RIPA lysis buffer (Solarbio, China) supplemented with a protease inhibitor (YaMei China). Protein concentration was quantified via the BCA Protein Assay Kit (Pierce™, Thermo Fisher Scientific). Equal amounts of protein were resolved on 8-12% gradient SDS-PAGE gels and transferred to PVDF membranes using a semi-dry transfer system (Bio-Rad). Membranes were blocked with 5% non-fat milk in TBST for 1 h at room temperature, followed by incubation with primary antibodies at 4°C overnight: MMP11 (1:600, #30615-1-AP, Proteintech), E-cadherin (1:40,000, #20874-1-AP, Proteintech), Vimentin (1:50,000, #10366-1-AP, Proteintech), and GAPDH (1:200,000, #60004-1-Ig, Proteintech). After washing, membranes were incubated with HRP-conjugated anti-rabbit IgG secondary antibody (1:10,000, #SA00001-2, Proteintech) for 2 h at room temperature. Protein bands were visualized using ECL Plus Substrate (Bio-Rad) and quantified using Image Lab™ Software (v6.1, Bio-Rad).

### Immunohistochemistry

Archived pathological tissue specimens were obtained from 5 patients with benign prostatic hyperplasia and 5 patients with prostate adenocarcinoma at the Affiliated Hospital of Guizhou Medical University. Immunohistochemical staining was performed on paraffin-embedded tissue sections using an automated immunostainer (DAKO, Denmark) with a rabbit monoclonal anti-MMP11 antibody (1:300 dilution, #30615-1-AP, Proteintech). Staining intensity was analyzed in three independent random fields and independently evaluated by two experienced pathologists. This study was approved by the Ethics Committee of the Affiliated Hospital of Guizhou Medical University (Approval No. 2024-91).

### EDU proliferation test

The 5-ethynyl-2′-deoxyuridine (EdU) Cell Proliferation Kit was obtained from Beyotime Biotechnology (Shanghai, China). DU145 cells were seeded into 24-well plates at a density of 3 × 10³ cells/well and cultured until reaching 70–80% confluency. Cells were pulse-labeled with EdU working solution (20 μM, Servicebio, China) for 2 hours at 37°C in 5% CO_2_, followed by fixation with 4% paraformaldehyde (PFA) for 15 minutes and permeabilization with 0.5% Triton X-100 for 20 minutes. Proliferating cells were stained using the Click-iT™ EdU Alexa Fluor™ 594 Imaging Kit (Thermo Fisher Scientific) according to the manufacturer’s protocol. Fluorescent images were captured using an inverted fluorescence microscope (Nikon Eclipse Ts2R-FL) at 20 × magnification.

### Cell scratch test

Plasmid-transfected DU145 cells were cultured in 6-well plates until reaching 100% confluency in complete growth medium (RPMI-1640 + 10% FBS). A standardized wound was created by scraping the monolayer with a 200 μl sterile pipette tip, followed by three washes with PBS to remove detached cells. Cells were maintained in serum-free medium (RPMI-1640 + 1% FBS) to minimize proliferation bias. Wound closure was monitored at 0/48 hours post-scratching using an inverted phase-contrast microscope (Nikon Eclipse Ts2R-FL, 10× objective). Migration rates were quantified by measuring the residual wound area with ImageJ software (v1.53t).

### Transwell invasion experiment

Pre-chill pipette tips and plates on ice. Dilute NEST Matrigel with a serum-free medium at a 1:11 ratio (Matrigel: medium) on ice. Coat the upper chamber of 8 μm pore Transwell inserts (NEST, China) with 100 μL diluted Matrigel and incubate at 37°C for 1h to allow polymerization. Remove excess liquid and rehydrate inserts with serum-free medium for 30 min. Resuspend transfected DU145 cells in serum-free medium and seed 2 × 10^4^ cells/well into the upper chamber (200 μL/well). Add 500 μL of complete medium (RPMI-1640 + 10% FBS) to the lower chamber as a chemoattractant. Incubate for 24 h at 37°C in 5% CO_2_. Remove non-invading cells from the upper chamber with a cotton swab. Fix invaded cells with 4% paraformaldehyde (15 min), stain with 0.1% crystal violet (20 min), and capture five random fields per insert under an inverted microscope (Nikon Eclipse Ts2R-FL, 10× objective). Quantify invasion using ImageJ software (v1.53t) by counting crystal violet-positive cells.

### Statistical analysis

All statistical analyses were performed using R software (version 4.2.2). Gene expression differences or pathway enrichment scores were assessed using the nonparametric Wilcoxon rank-sum test and parametric paired Student’s t-test, with statistical significance defined as a two-sided P < 0.05. Pearson correlation coefficients (r) were calculated to quantify linear associations between variables. Correlations were considered biologically meaningful if they met both thresholds: |r| ≥ 0.2 and P < 0.05. Survival analysis (disease-free survival, DFS) was conducted using Cox proportional-hazards regression and Kaplan-Meier curves with log-rank tests.

## Result

### Clinical relevance of MMPs

**Figure 1:**
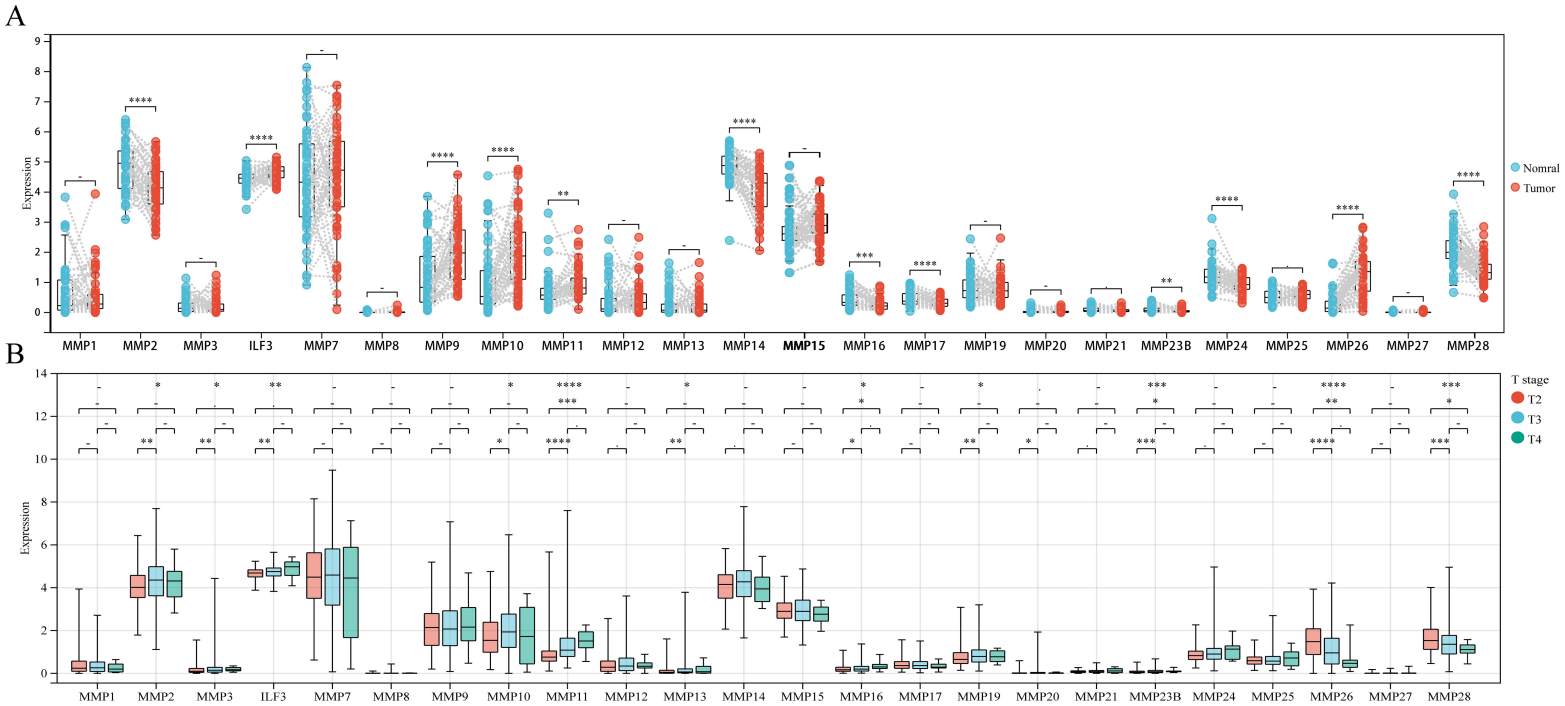
Differential expression of MMPs and its relationship with T staging. **(A)** Differential expression of 24 MMPs; **(B)** The expression levels of 24 MMPs in different T stages. ->0.05, *P< 0.05, **P< 0.01, ***P< 0.001, ****P< 0.0001.

**Figure 2:**
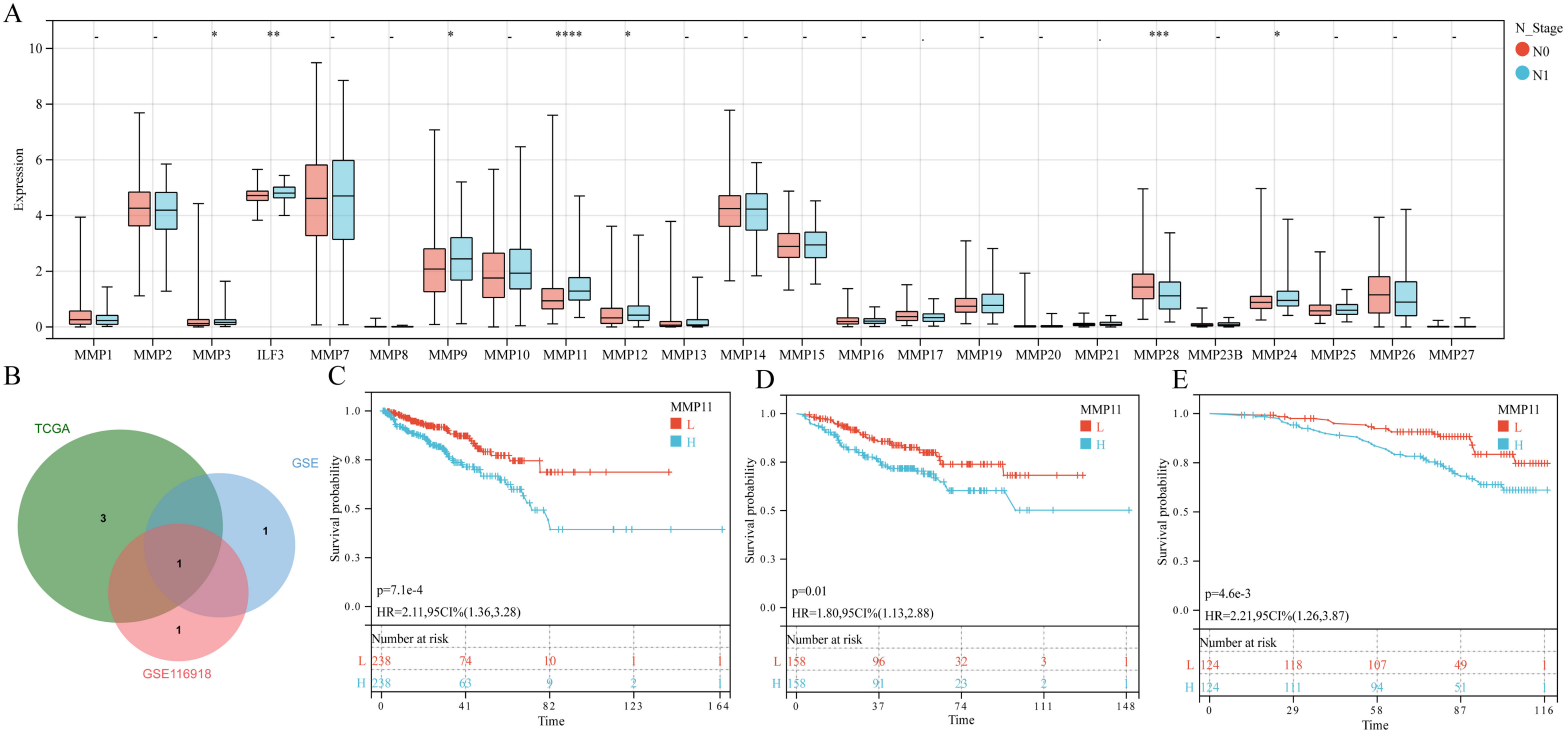
The relationship between MMPs and N staging, as well as prognostic characteristics. **(A)** The expression levels of 24 MMPs in different N stages; **(B)** Common prognostic feature genes; **(C-E)** K-M curves of MMP11 and DFS in three queues, **(C)** TCGA-PRAD; **(D)** GSE; **(E)** GSE116918. ->0.05, *P< 0.05, **P< 0.01, ***P< 0.001, ****P< 0.0001, L, Low expression group; H, High expression group.

The paired sample comparison graph (**Figure 1A**) demonstrated that the expression levels of ILF3, MMP9, MMP10, MMP11, and MMP26 in PCa tumor tissue were elevated than adjacent non-tumor tissues (P < 0.05). Conversely, MMP2, MMP14, MMP16, MMP17, MMP23B, MMP24, and MMP28 were reduced (P < 0.05), while the remaining MMPs remained unchanged (P > 0.05). Similarly, the boxplot (**Figure 1B**) showed that MMP2, MMP3, ILF3, MMP11, MMP13, MMP16, MMP19, MMP20, and MMP23B expression were upregulated in T3 and/or T4 stages compared to T2 (P < 0.05), whereas MMP28 and MMP26 expression was downregulated (P < 0.05), while no significant difference was observed for other MMPs (P > 0.05). Furthermore, the boxplot (**Figure 2A**) illustrated that MMP3, ILF3, MMP9, MMP11, MMP12, and MMP24 expression in N1 stage was upregulated than N0 stage, while MMP28 expression was downregulated (P < 0.05) and the remaining MMPs were similar between the two groups (P > 0.05). To further elucidate the prognostic significance of MMPs, the univariate Cox-regression analysis was performed to investigate the relationship between MMPs and DFS of PCa in three cohorts of PCa patient. The result suggested that (**Figures 2B-E**) only MMP11 was associated with shorter DFS (P < 0.05). Consequently, MMP11 was selected as the primary marker for subsequent analysis.

### The effect of MMP11 on the immune microenvironment in PCa

**Figure 3:**
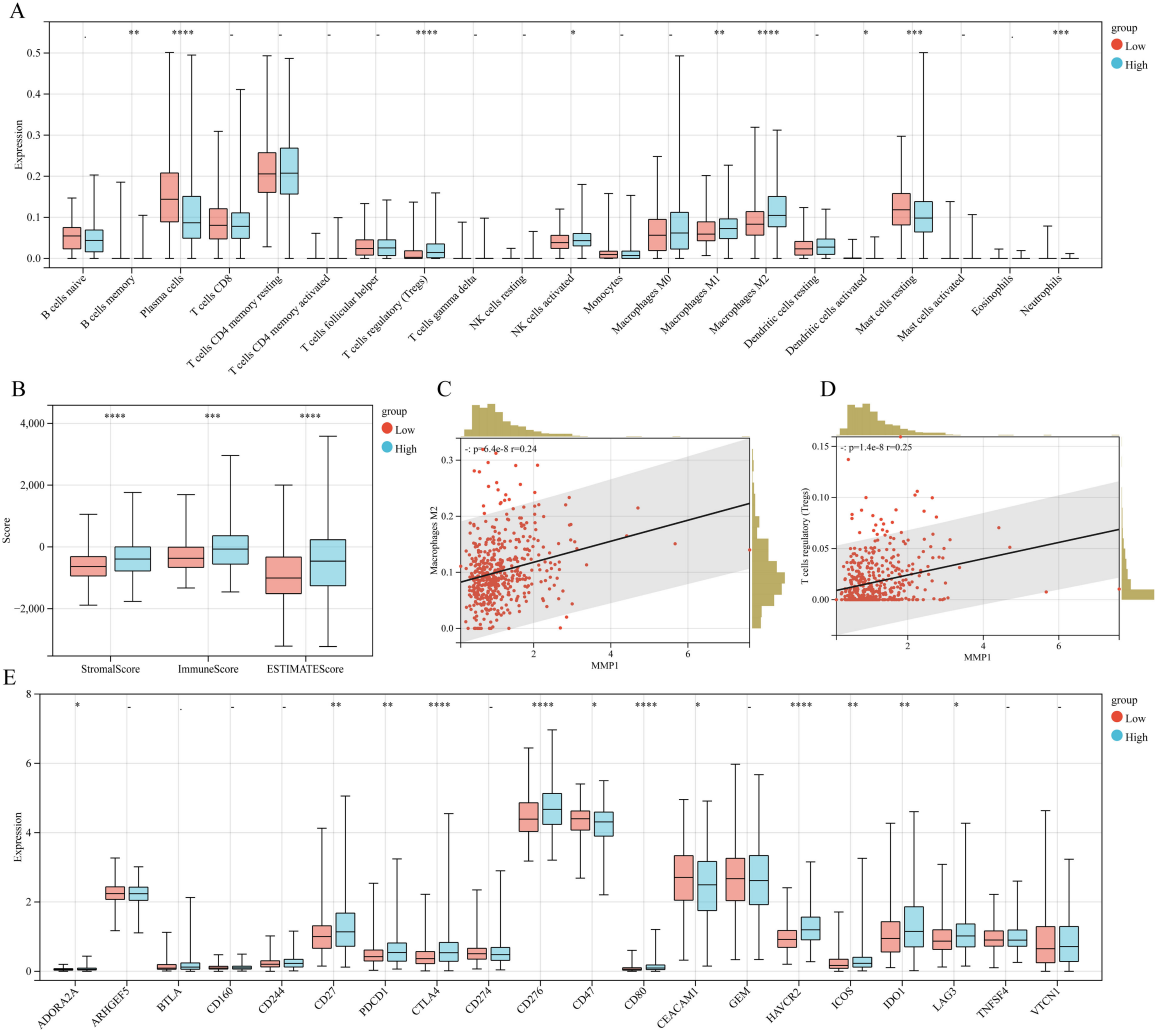
The relationship between MMP11 and immune microenvironment. **(A)** Comparison of infiltration degree of 21 immune cells in MMP11 high and low expression groups; **(B)** Comparison of immune infiltration scores between MMP11 high and low expression groups; **(C)** The relationship between MMP11 and M2 macrophages; **(D)** The relationship between MMP11 and Treg; **(E)** Comparison of infiltration degree of 20 common immune checkpoint genes in MMP11 high and low expression groups. ->0.05, *P< 0.05, **P< 0.01, ***P< 0.001, ****P< 0.0001.

Given the intimate association between TME and cancer progression, we utilized CIBERSORT to assess the immune-cell infiltration degree which showed that the Tregs, activated NK cells, and macrophage M1/M2 exhibited greater infiltration levels in the MMP11-high expression group than MMP11-low expression group, while the plasma cells and resting mast cells displayed lower infiltration levels (**Figure 3A**). These were subsequently corroborated by ESTIMATE analysis, that high expression group exhibited elevated immune-score, estimate-score, and stromal-score (**Figure 3B**). Moreover, the Pearson-correlation analysis (**Figures 3C, D**) demonstrated a positive correlation between MMP11 expression level and infiltration degree of macrophages M2 (r = 0.24, P = 6.4e-08) and Tregs (r = 0.25, P = 1.4e-08). A deeper analysis (**Figure 3E**) further uncovered that the MMP11-high expression group exhibited more extensive immune checkpoints expression. Notably, among the 20 commonly observed immune checkpoints, 10 genes (ADORA2A, CD27, PDCD1, CTLA4, CD276, CD80, HAVCR2, ICOS, IDO1, and LAG3) were significantly higher in the MMP11-high expression group. Meanwhile, correlation analysis showed that (**Supplementary Figure S1**) MMP11 was positively correlated with the expression of most immunomodulatory genes (including chemokines, chemokine receptors, major histocompatibility complexes (MHC), co-stimulatory factors, and co-inhibitory factors). These data not only suggested that MMP11 has significant impacts on the immune microenvironment of PCa, but may also influenced the efficacy of its immunotherapy. As a consequence, we further calculated to assess the Easier score, that the MMP11-high expression group exhibited significantly higher Easier score (**Supplementary Figure S2A**), which underscored the likelihood of deriving more benefits from immunotherapy.

### MMP11 was associated with higher tumor mutation burden in PCa

Elevated tumor neoantigen burden is strongly associated with favorable immunotherapy outcomes. Given the established links between neoantigens, tumor mutational burden (TMB), and microsatellite instability (MSI) (15, 16), we observed significantly higher TMB and MSI in the MMP11-high group (**Supplementary Figures S2B, C**), suggesting enhanced potential for immunotherapy responsiveness. To evaluate MMP11’s specific impact on ICB efficacy, we analyzed two independent anti-PD-1-treated cohorts. MMP11-high patients exhibited a trend toward prolonged overall survival (OS) in both melanoma (HR = 0.62, 95% CI 0.37–1.04, P = 0.07) and clear cell renal carcinoma (HR = 0.64, 95% CI 0.33–1.31, P = 0.19) cohorts (**Supplementary Figures S2D, E**). Furthermore, mutational profiling revealed an enrichment of high-risk driver mutations in the MMP11-high group, including TP53 (17%), SPOP (13%), TNT (11%), and KMT2D (7%) (**Supplementary Figures S3A, B**), consistent with its association with poorer prognosis.

### Effect of MMP11 on PCa cells

**Figure 4:**
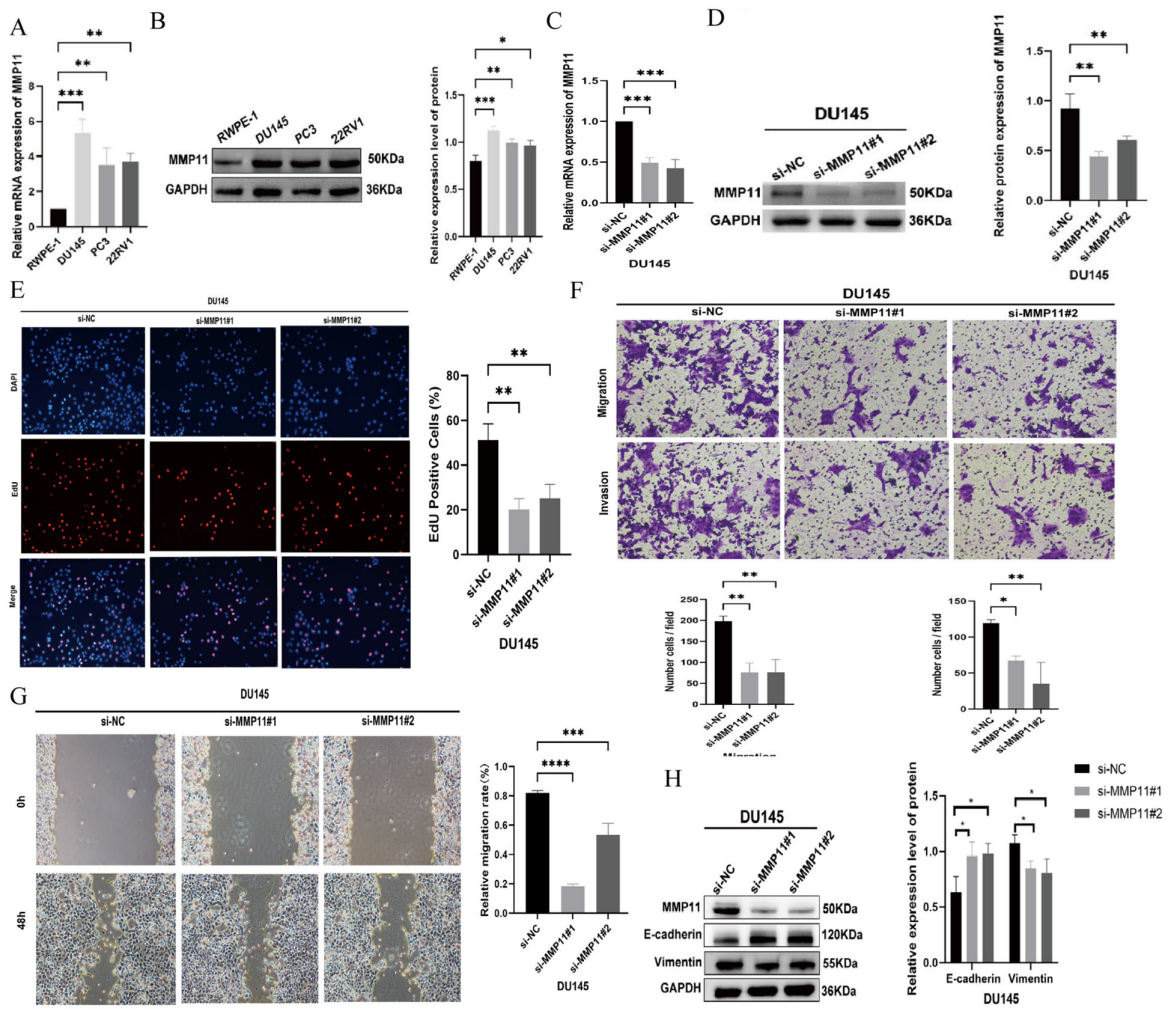
The impact of MMP11 on the biological behavior of PCa cells. **(A, B)** Expression validation of MMP11, **(A)** mRNA; **(B)** protein. **(C, D)** Knockout efficiency verification of MMP11, **(C)** mRNA; **(D)** protein. **(E)** Edu experiment detects changes in cell proliferation ability of DU145 cell line after knocking down MMP11; **(F)** Transwell migration assay detecting changes in cell migration and invasion ability of DU145 cell line after knocking down MMP11; **(G)** Scratch experiment detection of changes in cell migration ability of DU145 cell line after knocking down MMP11; **(H)** Changes in EMT marker proteins of prostate cancer cells after a decrease in MMP11 levels. ->0.05, *P< 0.05, **P< 0.01, ***P< 0.001, ****P< 0.0001.

qRT-PCR and Western blot analyses demonstrated elevated MMP11 mRNA and protein levels in prostate cancer (PCa) cell lines (DU145, PC3, 22RV1) compared to normal prostate epithelial cells (RWPE-1) (**Figures 4A, B**). Consistently, immunohistochemistry revealed significantly higher MMP11 expression in PCa patient tissues versus benign controls (**Supplementary Figure S4**). siRNA-mediated MMP11 knockdown in DU145 cells (**Figures 4C, D**) confirmed successful silencing. Functional assays revealed that MMP11 suppression significantly inhibited proliferation (EdU assay), invasion (Transwell assay), and migration (scratch healing assay) (**Figures 4E-G**), supporting its pro-tumorigenic role in PCa. To explore underlying mechanisms, ssGSEA of 50 oncogenic pathways identified epithelial-mesenchymal transition (EMT) activation in MMP11-high groups (**Supplementary Figure S5**). Subsequent analysis of EMT markers showed increased E-cadherin and decreased vimentin expression in MMP11-suppressed DU145 cells (**Figure 4H**), confirming that MMP11 knockdown attenuates EMT progression.

### Single cell atlas of PCa

**Figure 5:**
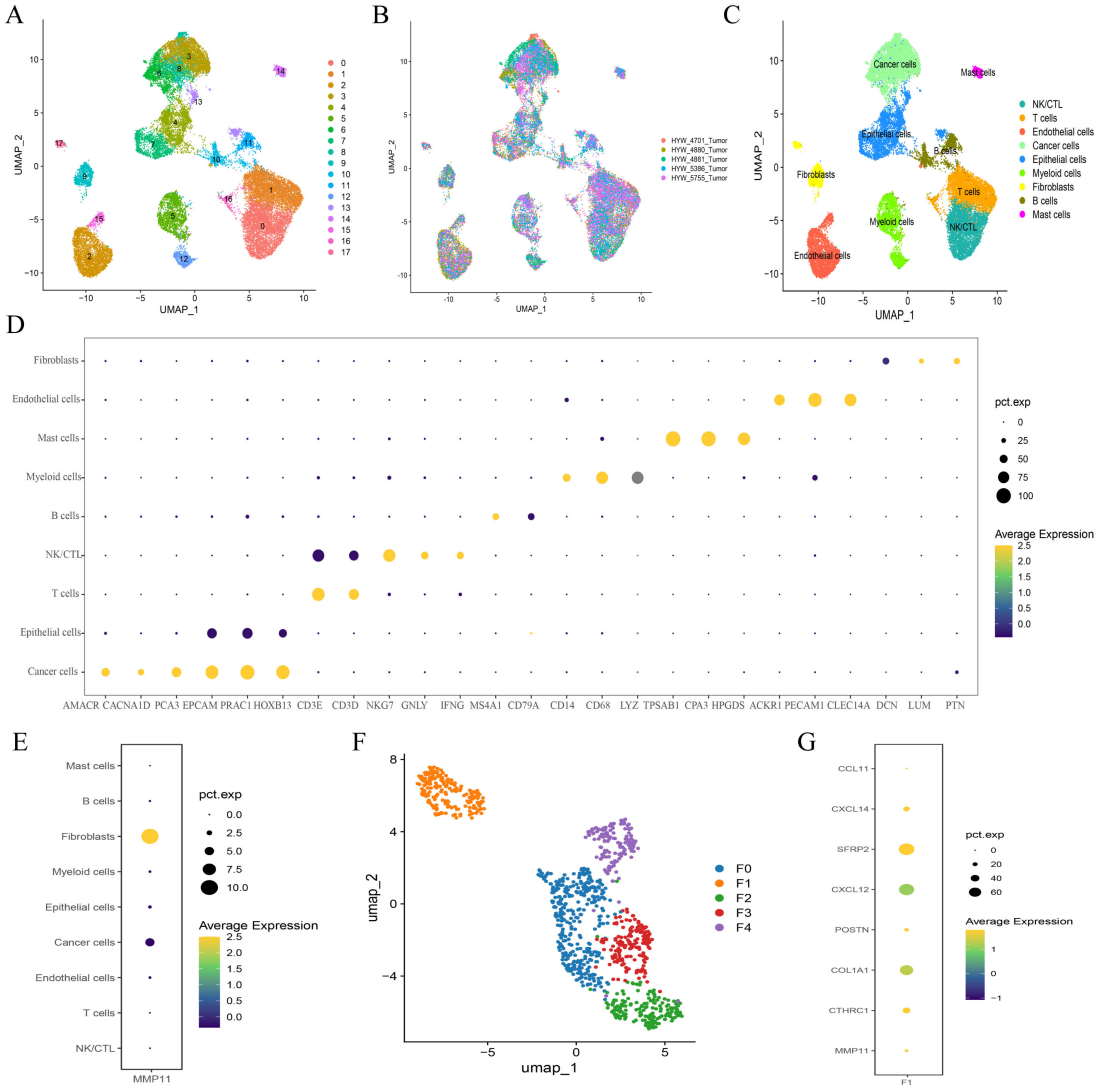
Single cell atlas of prostate cancer. **(A-C)** UMAP maps of all single cells, color coded as **(A)** 18 subcellular populations; **(B)** Different organizational origins; **(C)** 9 annotated subcellular populations; **(D)** Cell subpopulation annotation marker bubble plot; **(E)** Expression of MMP11 in 9 cell subpopulations; **(F)** Subgrouping of fibroblasts; **(G)** Expression of MMP11 in 5 fibroblast cells.

After rigorous quality control, we successfully obtained 28,564 cells and unambiguously distinguished them based on their classical markers, including 4,673 T cells (CD3D+, CD3E+), 4,393 NK/CTL cells (CD3D+, CD3E+, NKG7+, GNLY+, IFNG+), 4,137 normal epithelial cells (EPCAM+, PRAC1+, HOXB13+), 6,155 tumor cells (AMACR+, CACNA1D+, PCA3+), 3,548 endothelial cells (ACKR1+, PECAM1+, CLEC14A+), 1,441 B cells (MS4A1+, CD79A+), 1,112 fibroblasts (DCN+, LUM+, PTN+), 2,725 myelocytes (CD14+, CD68+, LYZ+), and 420 mast cells (TPSAB1+, CPA3+, HPGDS+) (**Figures 5A-D**). We also observed that MMP11 was predominantly expressed in fibroblasts which was corroborated with previous findings (**Figure 5E**). As a result, we categorized fibroblasts into distinct subpopulations and found that fibroblasts were subdivided into five heterogeneous cell clusters (designated as F0-F4) (**Figure 5F**). Moreover, the gene expression profile indicated that MMP11 was primarily localized within the F01 cell population (**Figure 5G**), while this cell population exhibited characteristics of angiogenesis (CXCL14, POSTN, SFRP2, CXCL12) and a matrix-immune suppressive phenotype (COL1A1, CTHRC1, CCL11) (**Figure 5G**); these suggested a potential association between F01 and PCa progression.

### MMP11(+) fibroblast promoted progression of PCa

**Figure 6:**
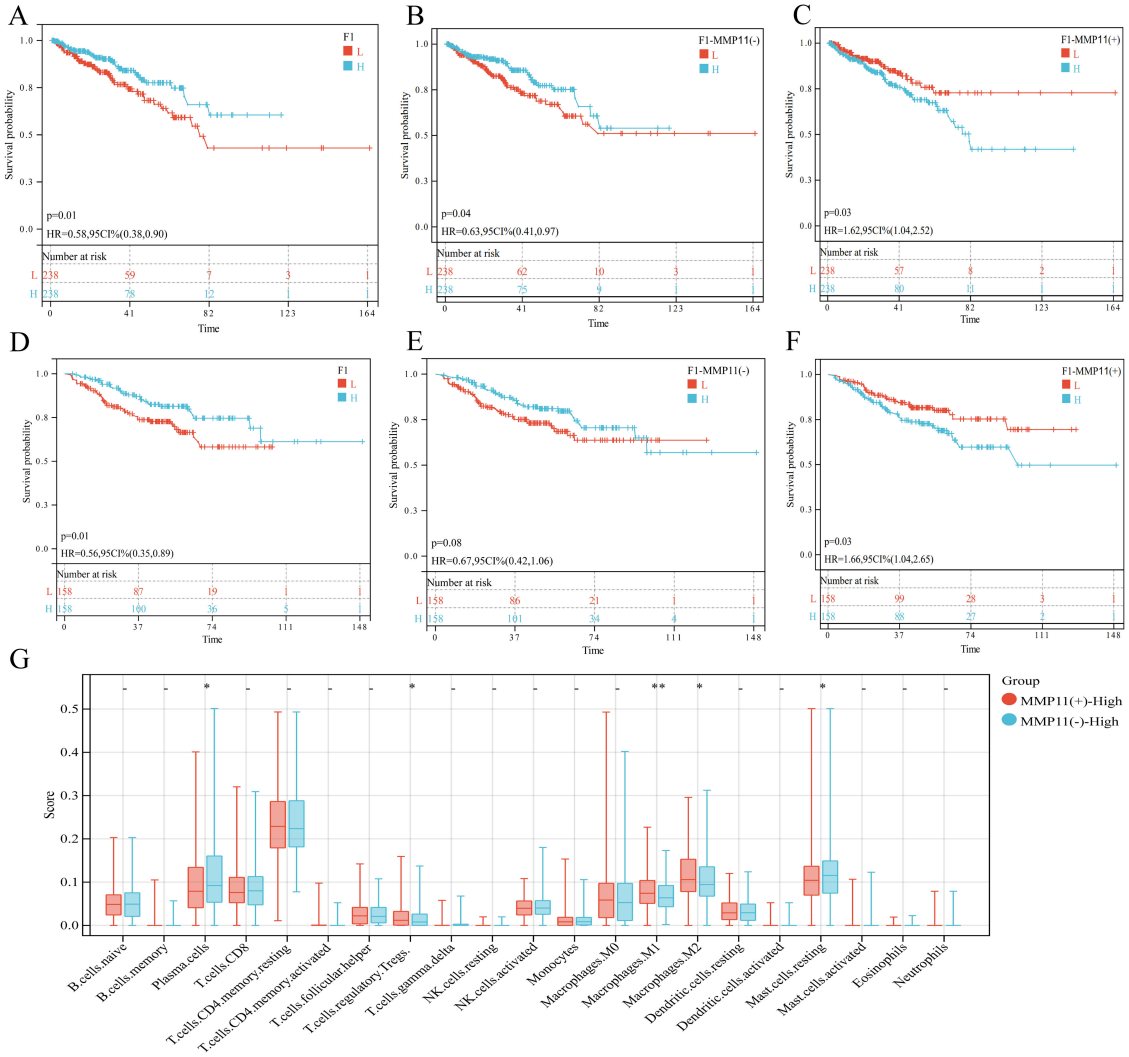
The effect of F01 on PCa. **(A-C)** The impact of three subpopulations of fibroblasts on the prognosis of PCa in the TCGA-PRAD cohort, **(A)** F01; **(B)** MMP11(-)F01; **(C)** MMP11(+)F01. **(D-F)** The impact of three subpopulations of fibroblasts on the prognosis of PCa in the GSE cohort, **(D)** F01; **(E)** MMP11(-)F01; **(F)** MMP11(+)F01. **(G)** Comparison of infiltration degree of 21 immune cells in MMP11 (-) F01 and MMP11 (+) F01. ->0.05, *P< 0.05, **P< 0.01.

**Figure 7:**
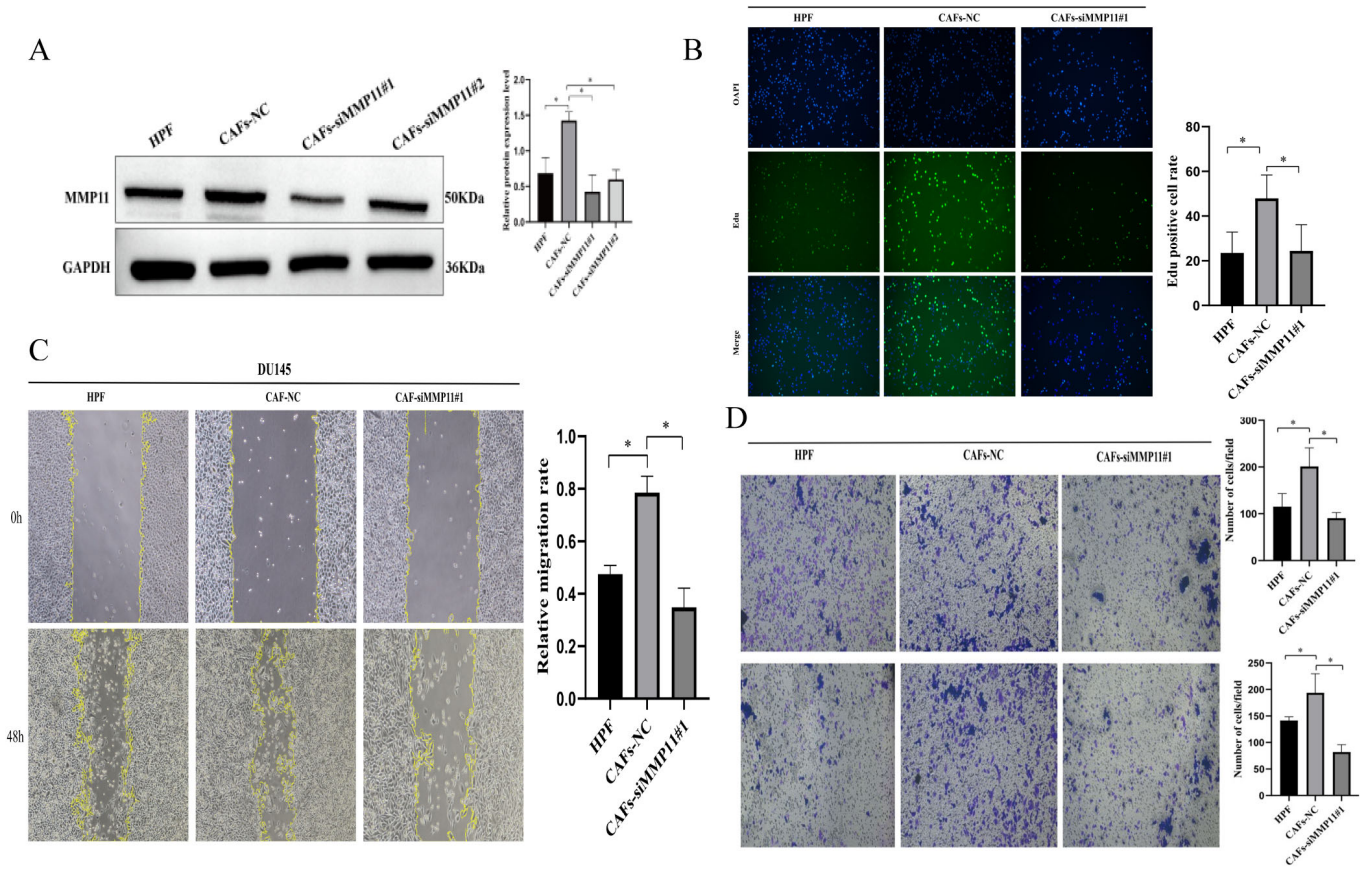
The pro tumorigenic effect of MMP11 induced tumor associated fibroblasts. **(A)** Protein expression levels of MMP11 in fibroblasts treated with different methods. **(B-D)** Suppression of MMP11 Expression in Cancer-Associated Fibroblasts (CAFs) Attenuates Their Pro-Tumorigenic Effects. **(B)** Edu experiment, **(C)** Scratch experiment, **(D)** Transwell experiment. *P< 0.05.

To assess the prognostic role of fibroblast subset F01 in PCa, we calculated F01 infiltration scores in the TCGA-PRAD and GSE cohorts. Kaplan-Meier curves revealed that higher F01 infiltration levels were paradoxically associated with prolonged DFS (**Figures 6A, D**), contradicting our initial hypothesis. Since only a small fraction of F01 cells expressed MMP11, we hypothesized that MMP11(+) fibroblasts specifically drive tumor progression. We stratified F01 into MMP11(+) and MMP11(−) subpopulations and recalculated their infiltration scores. Kaplan-Meier analysis showed that high MMP11(−) F01 infiltration remained associated with longer DFS (**Figures 6B, E**), whereas high MMP11(+) F01 infiltration correlated with shorter DFS (**Figures 6C, F**). Pathway enrichment analysis further demonstrated that MMP11(+) F01 cells exhibited activation of pro-tumorigenic and immunosuppressive pathways (IL-2, angiogenesis, PI3K-AKT-mTOR, and TGFβ), while MMP11(−) F01 cells enriched anti-cancer and immunostimulatory pathways (IFN-α/γ) (**Supplementary Figure S3C**). Western blot confirmed MMP11 upregulation in tumor-associated fibroblasts (CAFs) induced by conditioned medium compared to normal prostate fibroblasts. (**Figure 7A**). siRNA-mediated MMP11 knockdown in CAFs attenuated their pro-tumorigenic effects on DU145 cells, suppressing proliferation (EdU assay), migration (scratch assay), and invasion (Transwell assay) (**Figures 7B-D**). These pieces of evidence emphasize the association between MMP11 and the pro-tumorigenic phenotype transition of fibroblasts.

### MMP11(+) fibroblast promoted the formation of immunosuppressive microenvironment in PCa

**Figure 8:**
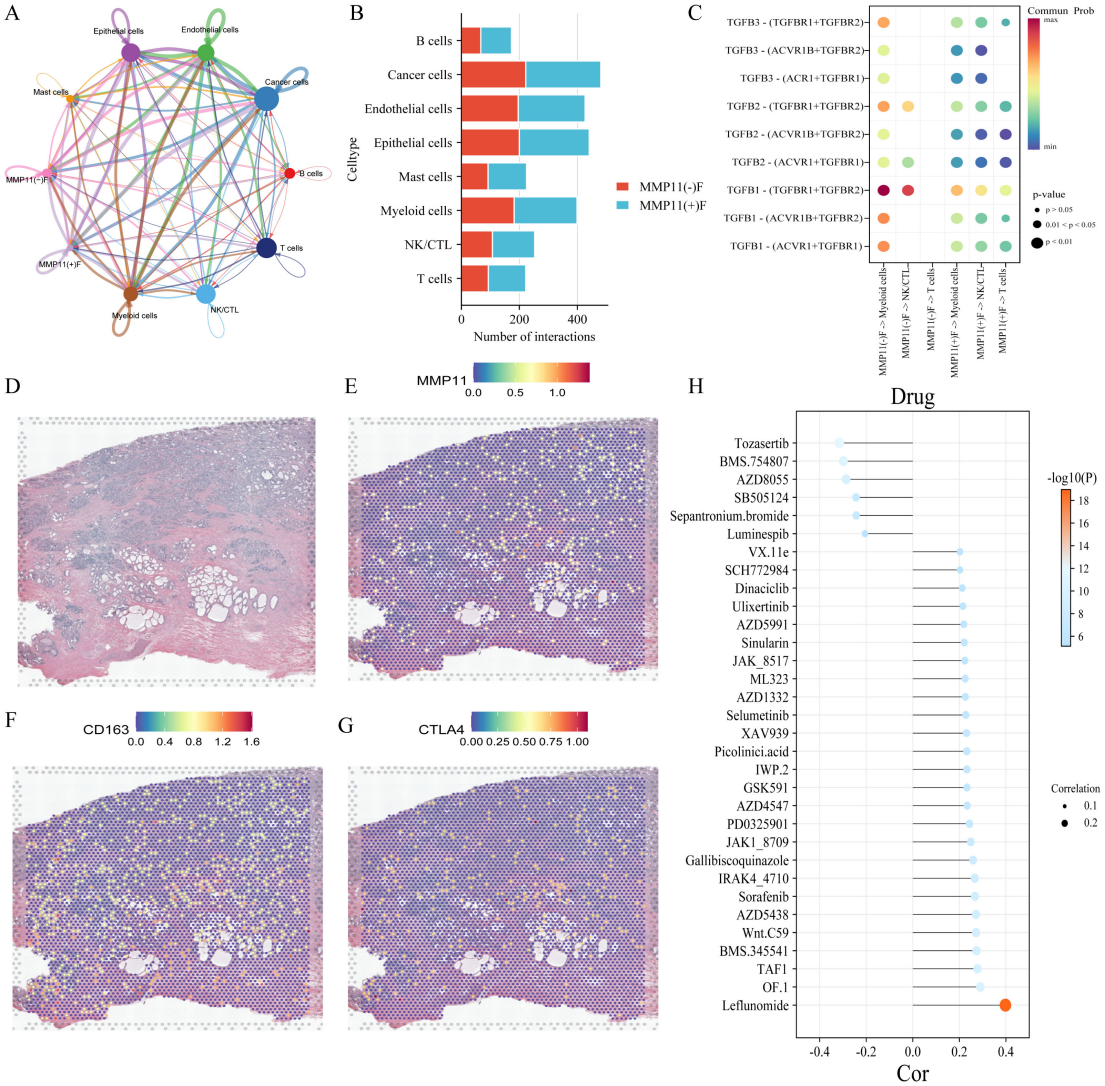
The impact of MMP11 (+) F01 on the immune microenvironment and the effect of MMP11 on drug sensitivity. **(A)** interaction of various cell types in the microenvironment of PCa; **(B)** Comparison of the number of MMP11(+)F01 and MMP11(-)F interactions with other cell types; **(C)** interaction of MMP11(+)F01 and MMP11(-)F with T cells, NK/CTL cells, and myeloid cells via TGFβ-related receptor-ligand pairs; **(D-G)** spatial positioning situation; **(H)** the correlation between MMP11 and sensitivity to 32 anti-tumor drugs.

To investigate the impact of MMP11(+)F01 on microenvironment, we conducted a comparative analysis on the infiltration levels of immune-cells between MMP11(-)F01 high infiltration group and MMP11(+)F01 high infiltration group. The MMP11(+)F01 high infiltration group exhibited more infiltration of immune suppressive cells (Tregs and macrophages M2) than MMP11(-)F01 group (**Figure 6G**). Meanwhile, Pearson correlation analysis revealed a positive correlation between MMP11(+) F01 cells and Tregs (R = 0.20, P = 7.1e-06) and macrophages M2 (R = 0.27, P = 3.0e-09) (**Supplementary Figures S3D, E**). We further analyzed the communication of MMP11(+) F01 with other cell types in the microenvironment of PCa and showed that MMP11(+) F01 not only communicated more intensively with other cell types compared to MMP11(-) fibroblasts but also affected T cells, NK/CTL, and myeloid cells through a greater number of TGFβ-associated receptor-ligand pairs (**Figures 8A-C**). However, previous studies have demonstrated that the TGFβ pathway normally acts as a key pathway for fibroblasts to induce the formation of promote-tumor immune cells from anti-tumor immune cells (17, 18). Similarly, spatial transcriptomic data showed that MMP11 overlaped in spatial location with a marker for M2 macrophages (CD163) and a marker for Tregs (CTLA4) (**Figures 8D-G**). These evidences revealed the crucial role of MMP11 (+) F01 in the formation of the PCa immune suppressive microenvironment.

### The effect of MMP11 for drug sensitivity on PCa

To further elucidate the effect of MMP11 for drug sensitivity on PCa, we calculated IC50s for 198 drugs covering various chemotherapeutic, anti-vascular, and targeted agents for all patients in the TCGA-PRAD cohort. Our findings demonstrate that MMP11 is associated with reduced sensitivity to 26 drugs, most strongly with leflunomide (R=0.40, P=1.08×10^-^¹^9^), OF.1 (R=0.29, P=8.82×10^-^¹¹), and TAF1_5496 (R=0.28, P=5.57×10^-^¹¹). Conversely, MMP11 correlates with enhanced sensitivity to six agents: tozasertib (R=−0.32, P=1.47×10^-^¹²), BMS-754807 (R=−0.30, P=2.56×10^-^¹¹), AZD8055 (R=−0.29, P=1.64×10^-^¹^0^), SB505124 (R=−0.24, P=6.36×10^-8^), ipratropium bromide (R=−0.24, P=6.73×10^-8^), and luminbin (R=−0.21, P=5.65×10^-6^) (**Figure 8H**). These results indicate that MMP11 modulates therapeutic drug responsiveness in PCa.

### MMP11 is associated with poor prognosis in various tumors

**Figure 9:**
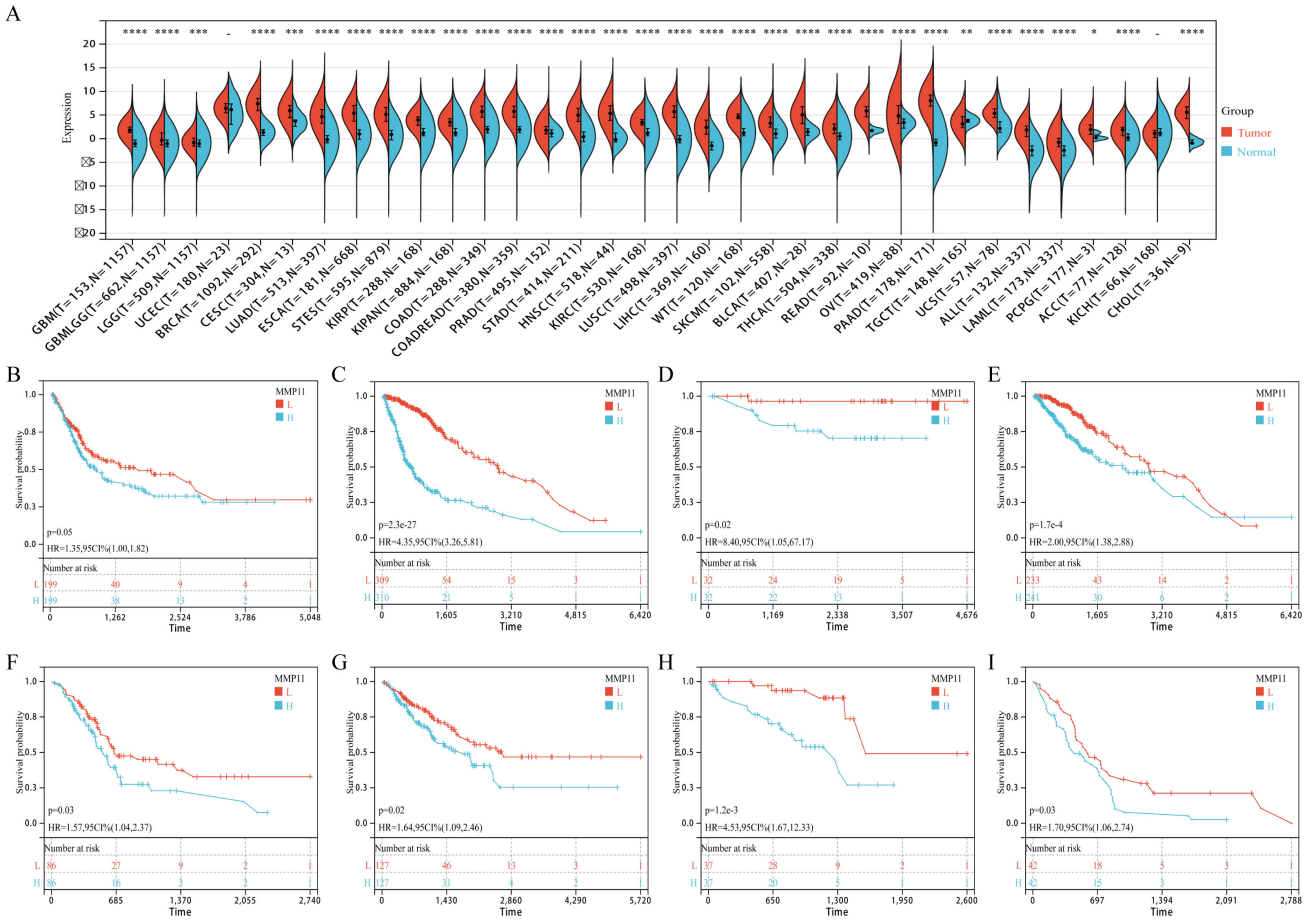
The expression of MMP11 in normal and malignant samples through using GTEx and TCGA datasets. **(A)** MMP11 expression in normal tissues based on the GTEx databases; **(B-I)** The relationship between MMP11 and overall survival of tumors, **(B)** BLCA, **(C)** GBMLGG, **(D)** KICH, **(E)** LGG, **(F)** PAAD, **(G)** SARC, **(H)** UVW, **(I)** MESO. ->0.05, *P< 0.05, **P< 0.01, ***P< 0.001, ****P< 0.0001.

To further investigate the potential clinical significance of MMP11, we examined its expression levels in various types of tumors and assessed its prognostic value. Compared with normal tissues, MMP11 was significantly higher in almost all tumors including glioblastoma multiforme (GBM), glioma (GBMLGG), brain lower grade glioma (LGG), breast carcinoma (BRCA), cervical squamous cell carcinoma and endocervical adenocarcinoma (CESC), lung adenocarcinoma (LUAD), esophageal carcinoma (ESCA), stomach and esophageal carcinoma (STES), kidney renal papillary cell carcinoma (KIRP), colon adenocarcinoma (COAD), colon adenocarcinoma/rectum adenocarcinoma esophageal carcinoma (COADREAD), prostate cancer (PRAD), stomach adenocarcinoma (STAD), head and Neck squamous cell carcinoma (HNSC), kidney renal clear cell carcinoma (KIRC), lung squamous cell carcinoma (LUSC), liver hepatocellular carcinoma (LIHC), Wilms tumor (WT), skin cutaneous melanoma (SKCM), bladder urothelial carcinoma (BLCA), thyroid carcinoma (THCA), rectum adenocarcinoma (READ), ovarian serous cystadenocarcinoma (OV), pancreatic adenocarcinoma (PAAD), uterine carcinosarcoma (UCS), acute lymphoblastic leukemia (ALL), acute myeloid leukemia (LAML), pheochromocytoma and paraganglioma (PCPG), adrenocortical carcinoma (ACC), kidney chromophobe (KICH), and cholangiocarcinoma (CHOL) (**Figure 9A**). On the contrary, MMP11 was significantly lower in testicular germ cell tumors (TGCT) (**Figure 9A**). Meanwhile, Kaplan-Meier curves showed that higher MMP11 expression was associated with shorter overall survival (OS) in diverse tumors including BLCA (**Figure 9B**), GBMLGG (**Figure 9C**), KICH (**Figure 9D**), LGG (**Figure 9E**), PAAD (**Figure 9F**), SARC (**Figure 9G**), UVW (**Figure 9H**), and mesothelioma (**Figure 9I**). These findings suggested that MMP11 had broad clinical value in predicting the prognosis of multiple tumors.

## Discussion

PCa stands as the most prevalent male cancer and leading the second most cancer-related deaths globally. Despite the treatment advance, the outlook remains grim for metastatic and castration-resistant PCa (1, 2). It was thus essential to identify reliable molecular markers to predict disease progression and recurrence to improve survive for PCa patients (19, 20). As a zinc-dependent endopeptidases family, the 24 MMPs played diverse roles in tumor metastasis, angiogenesis, immune evasion, and treatment resistance (9–11). Recent studies also hinted MMPs as potential prognostic biomarkers and therapeutic target for PCa (4–6, 11, 21, 22). However, most of them primarily focused on single or several MMPs in diverse cancer types, the comprehensive relationship between all MMPs and PCa remained further exploration.

In this study, the expression and prognostic significance of 24 MMPs in PCa was throughly assessed. We found that the majority MMPs exhibited differential expression patterns between tumor and normal tissues while certain MMPs expression was associated with PCa pathological features, which revealed the intricate relationship between MMPs and PCa. For the 24 MMPs, Cox-regression and K-M survival curve analysis presented that only elevated MMP11 consistently associated to shorter DFS for PCa patients across the three cohorts. We thus comprehensively analyzed MMP11 to explore its underlying role in PCa.

MMP11, also known as stromelysin-3, is an MMPs member that plays a pivotal role in ECM degradation and modification (23). MMP11 expression has been reported to be elevated in diverse tumors and correlated with unfavorable outcomes (24, 25). Our finding showed a similar trend that MMP11 expression was increased in PCa tissues and cell lines, while it was associated with unfavorable PCa survival. Meanwhile, MMP11 downregulation inhibited the proliferative, migratory, and invasive capabilities of PCa cells, underscoring its crucial role in PCa pathogenesis and progression. To further explore the underlying mechanisms, a functional enrichment analysis was performed and found that high expression of MMP11 was associated with the activation of EMT process in PCa. We then examined the alteration of EMT markers and found that MMP11 knockdown significantly upregulated E-cadherin and downregulated vimentin (two essential EMT markers), this suggested that MMP11 might promote PCa biological behavior through the inducing EMT process.

Emerging studies highlight MMP11’s regulatory role in the tumor microenvironment (TME) (26–28), which may contribute to PCa progression (28). The TME, composed of tumor cells and surrounding immune/stromal components, provides a critical niche for tumor survival and progression (29, 30). TME reprogramming has been shown to influence therapeutic responses and clinical outcomes (31, 32). We investigated MMP11’s impact on the PCa TME and observed that MMP11- high expression correlated with increased infiltration of Tregs and M2 macrophages—key immunosuppressive populations driving tumor progression, immune evasion, angiogenesis, and therapy resistance (33, 34). Although the precise mechanisms remain unclear, evidence suggests MMP11 may mediate fibroblast phenotypic switching (26–28, 35).

To investigate MMP11’s role in fibroblast-mediated remodeling of the PCa microenvironment, we integrated single-cell RNA sequencing and spatial transcriptomic data. Our results showed that MMP11(+) fibroblasts exhibited a pro-tumorigenic phenotype characterized by angiogenesis and immune tolerance compared to MMP11(-) fibroblasts, were spatially located more closely to macrophage M2s and Tregs, communicated more tightly with peripheral cells, and could affect T cells, NK/CTL, and myeloid lineage through more TGFβ-related receptor ligand pairs cells. Whereas, the TGFβ pathway to was shown to be critical for fibroblast-induced conversion of anti-tumor immune cells into tumor-promoting immune cells (17, 18). Similar, *in vitro* experiments confirmed elevated MMP11 expression in PCa-CAF compared to normal fibroblasts. siRNA-mediated MMP11 knockdown in CAFs attenuated their pro-tumorigenic effects on DU145 cells, suppressing proliferation These findings collectively highlight MMP11’s central role in reprogramming fibroblast phenotypes to foster an immunosuppressive, pro-angiogenic tumor microenvironment that drives PCa progression.

In addition, the androgen receptor (AR) signaling pathway is critical for PCa proliferation and survival, and androgen deprivation therapy (ADT) remains the cornerstone of PCa treatment (36, 37). Emerging evidence links specific CAFs subtypes to ADT failure, likely mediated by intricate crosstalk between CAFs and AR signaling (38–40). While CAFs exhibit low baseline AR signaling activity that promotes tumor progression independently of ADT (38), paradoxically, AR pathway inhibition in CAFs triggers compensatory cytokine secretion to sustain tumor growth (37). Notably, elevated expression of specific genes in CAFs may suppress AR signaling in tumor cells and exacerbate castration resistance (39, 40). Our data reveal a negative correlation between androgen signaling and MMP11 expression (**Supplementary Figures S5, S6A**). Pharmacogenomic profiling further demonstrated reduced sensitivity to bicalutamide in MMP11-high patients (**Supplementary Figure S6B**), suggesting that MMP11(+) CAFs may contribute to castration resistance. However, these omics-based findings require mechanistic validation to delineate MMP11’s role in driving therapeutic resistance.

Our findings further demonstrate that elevated MMP11 expression correlates with heightened expression of immunomodulatory genes, including chemokines, chemokine receptors, MHC molecules, co-stimulatory factors, and co-inhibitory molecules—features strongly associated with favorable responses to cancer immunotherapy (41, 42). Cancer immunotherapies, particularly ICBs, have revolutionized oncology by enabling durable immune-mediated tumor control (42, 43), with promising efficacy observed in PCa patients with predominant bone metastases (44). However, limited neoantigen availability restricts clinical benefits to a subset of patients, underscoring the need for novel therapeutic targets (45). Notably, prior studies report that MMP11 exhibits immunogenic properties *in vitro*, effectively triggering cytotoxic T lymphocyte (CTL)-mediated granzyme B secretion (46). MMP11-targeted mRNA vaccines reversed immune tolerance and conferred anti-tumor protection in murine colon adenocarcinoma models (47). In our study, MMP11-high tumors exhibited elevated TMB and MSI—biomarkers linked to increased neoantigen production (42, 48, 49) and enhanced immunotherapy responsiveness (49). Collectively, these data position MMP11 as a promising dual-functional target: Immunogenicity: Directly eliciting anti-tumor CTL responses; Neoantigen Enrichment: Enhancing TMB/MSI-driven immune recognition.

Despite the potential of MMP-11 and other MMPs as promising therapeutic targets (24, 50), early clinical trials of broad-spectrum MMP inhibitors (MMPIs) failed to demonstrate significant clinical benefits (51). The rationale for MMPI development relied on the assumption that MMPs predominantly drive malignant processes in cancer. Recent studies, however, suggest a need to reassess the role of MMPs in cancer, as evidence indicates they may also exert protective or context-dependent effects (52–54). Preclinical models often employ MMP inhibition early and continuously during tumor progression, yet clinical data reveal that only early-stage patients benefit significantly (51). These highlight the importance of refining treatment timing and patient selection criteria. Emerging evidence of crosstalk between MMPs and miRNAs suggests that dual-targeting therapies combining these molecules could represent a novel therapeutic strategy (55, 56). The severe side effects of broad-spectrum MMPIs underscore the urgency of developing targeted delivery systems, such as nanoparticles, to improve safety and efficacy. Beyond therapeutic applications, the prognostic significance of MMPs in cancer positions them as valuable biomarkers for risk stratification in clinical practice (50).

While our study highlights the critical role of MMP11 in PCa, several limitations should be acknowledged. First, although *in vitro* experiments confirmed MMP11’s influence on PCa cell behavior, further validation is required to elucidate its underlying molecular mechanisms in PCa progression. Second, our findings rely heavily on omics data and *in vitro* models; thus, the *in vivo* role of MMP11 in PCa remains unclear. Future studies should incorporate *in vivo* models (e.g., organoids) to validate its systemic effects. Furthermore, while we identified MMP11’s role in promoting pro-tumorigenic fibroblast phenotypic shifts, the mechanistic impact of MMP11(+) fibroblasts on the PCa microenvironment warrants deeper investigation. Finally, although our data suggest MMP11’s association with ICBs and ADT outcomes, these findings are primarily derived from omics analyses. Mechanistic studies are essential to clarify how MMP11 modulates therapeutic responses to ICBs and ADT.

## Conclusion

We comprehensively evaluated the expression of MMPs in PCa and its prognostic significance. Elevated MMP11 is an independent risk factor for poor DFS in PCa patients, whereas it may regulate PCa progression by affecting the EMT process, TME reprogramming, and phenotypic conversion of CAFs. All these suggest that MMP11 is a promising therapeutic target for PCa.

## Data Availability

The original contributions presented in the study are included in the article/**Supplementary Material**. Further inquiries can be directed to the corresponding authors.
