# Supplementary material for: Landscape analysis of matrix metalloproteinases reveals key prognostic markers for prostate cancer
Source: Front Immunol. 2025 Jun 18;16:1582992. doi: 10.3389/fimmu.2025.1582992 (PMC12213759; doi:10.3389/fimmu.2025.1582992)
Supplement: Supplementary file 1 [file DataSheet1.docx]

Table. S1 Sequence of siRNA

| si-RNA | Sequence（5′-3′） |
| --- | --- |
| si-NC | Forward 5’- CGACUUAUUAGAUGCGGCUAUTT-3’  Reverse 5’- AUAGCCGCAUCUAAUAAGUCGTT-3’ |
| si-MMP11#1 | Forward 5’-CCUAAAGGUAUGGAGCGAUGUTT-3’  Reverse 5’- ACAUCGCUCCAUACCUUUAGGTT-3’ |
| si-MMP11#2 | Forward 5’-GUUCUUCCAAGGUGCUCAGUATT-3’  Reverse 5’-UACUGAGCACCUUGGAAGAACTT-3’ |

Table. S2 Primer synthesis sequence

|  | Primer sequence |
| --- | --- |
| *MMP11* | Forward 5’- GCCCTAAAGGTATGGAGCGATGTG -3’  Reverse 5’- CAGTGGGTAGCGAAAGGTGTAGAAG-3’ |


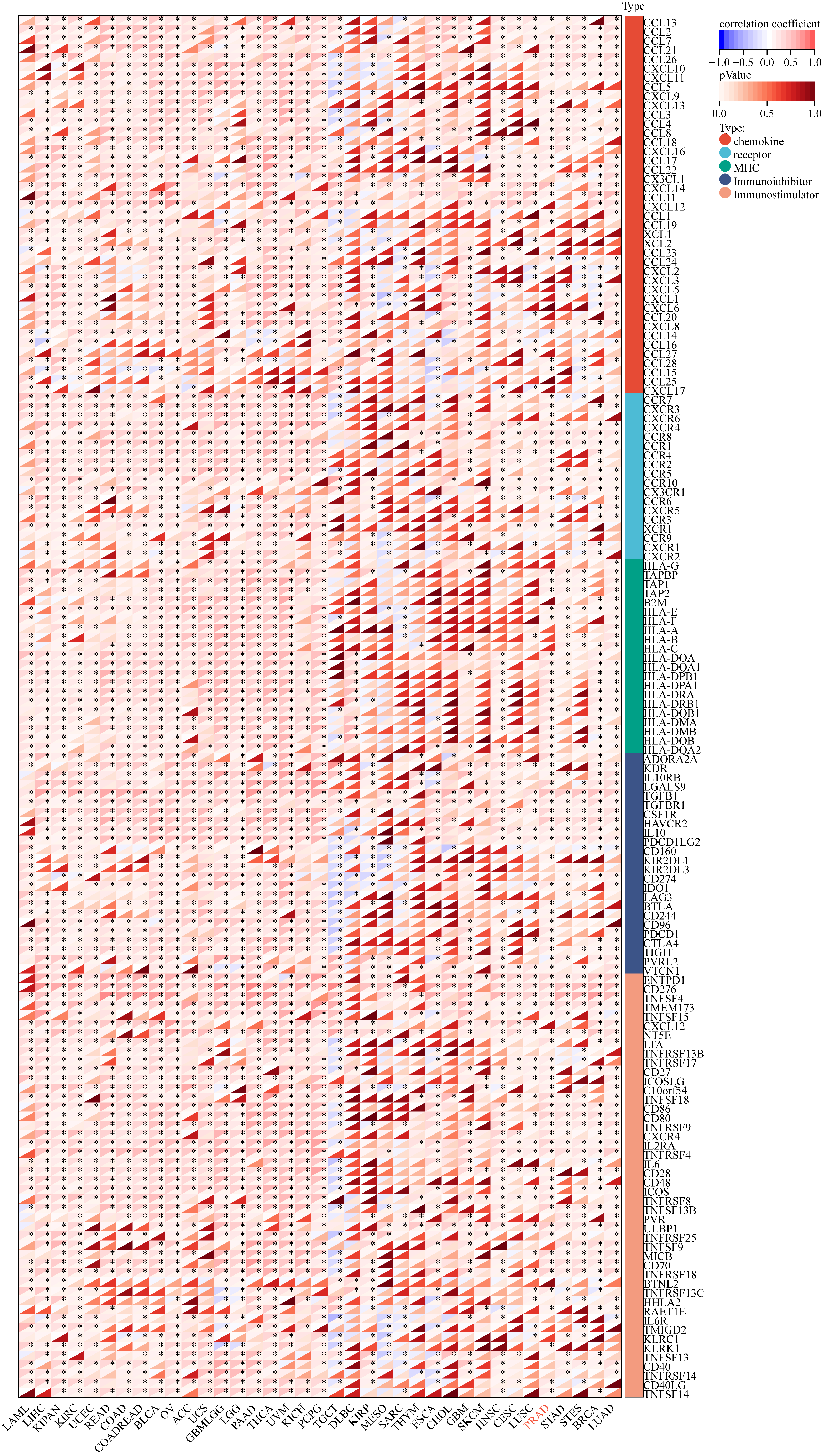


Fig S1: Correlation heatmap between MMP11 and immune regulatory genes

Note: *P< 0.05, **P< 0.01, ***P< 0.001, ****P< 0.0001


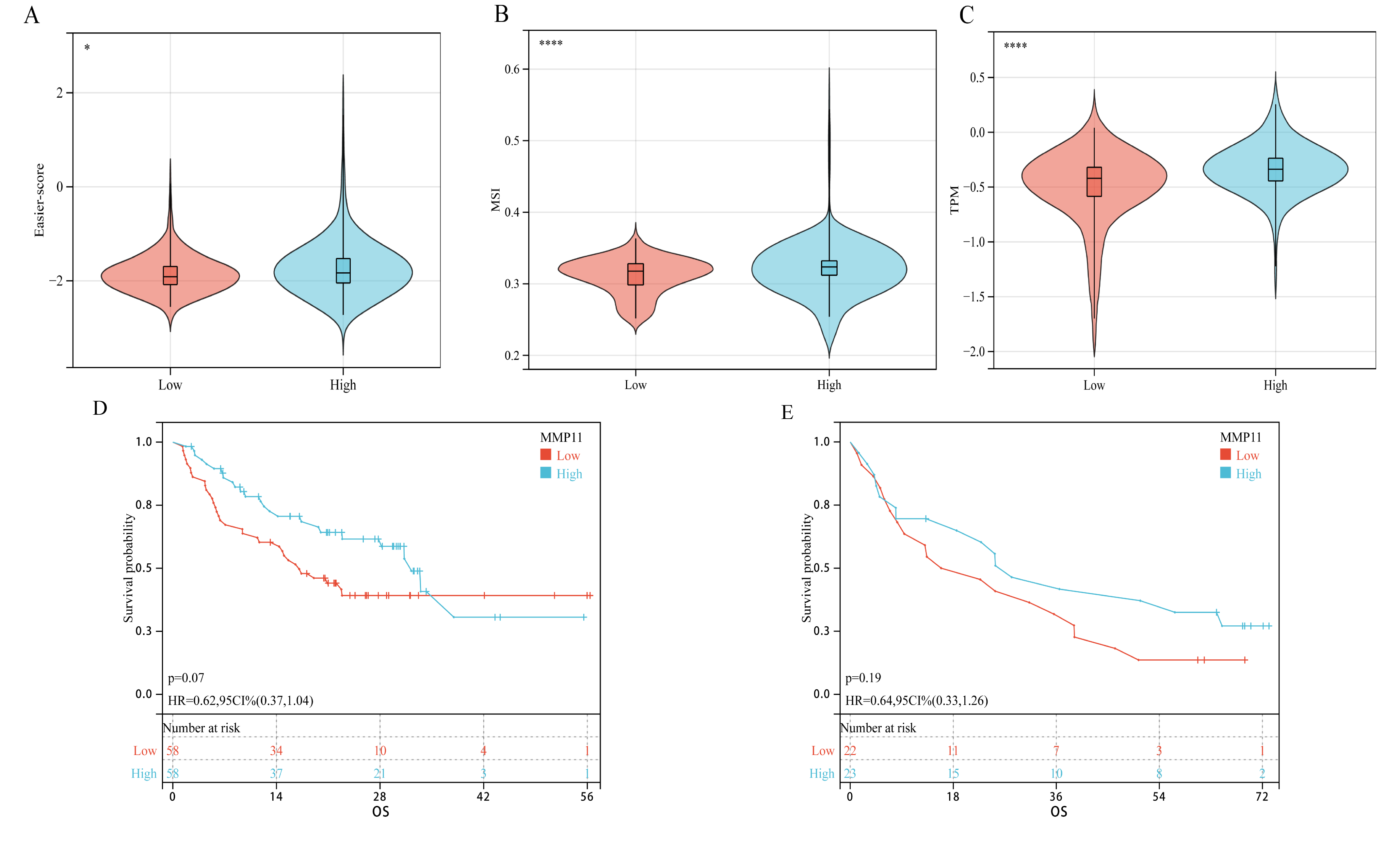


Fig.S2: The relationship and mutation burden between MMP11 and immunotherapy

A：Easier scores for MMP11 high and low expression groups; B：Microsatellite instability scores of MMP11 high and low expression groups; C: TMB of MMP11 high and low expression groups D: Overall survival of MMP11 high and low expression groups in melanoma cohort receiving PD-1/PD-L1 treatment; E: Overall survival of MMP11 high and low expression groups in Renal clear cell carcinoma cohort receiving PD-1/PD-L1 treatment

Note: -＞0.05, *P< 0.05, **P< 0.01, ***P< 0.001, ****P< 0.0001


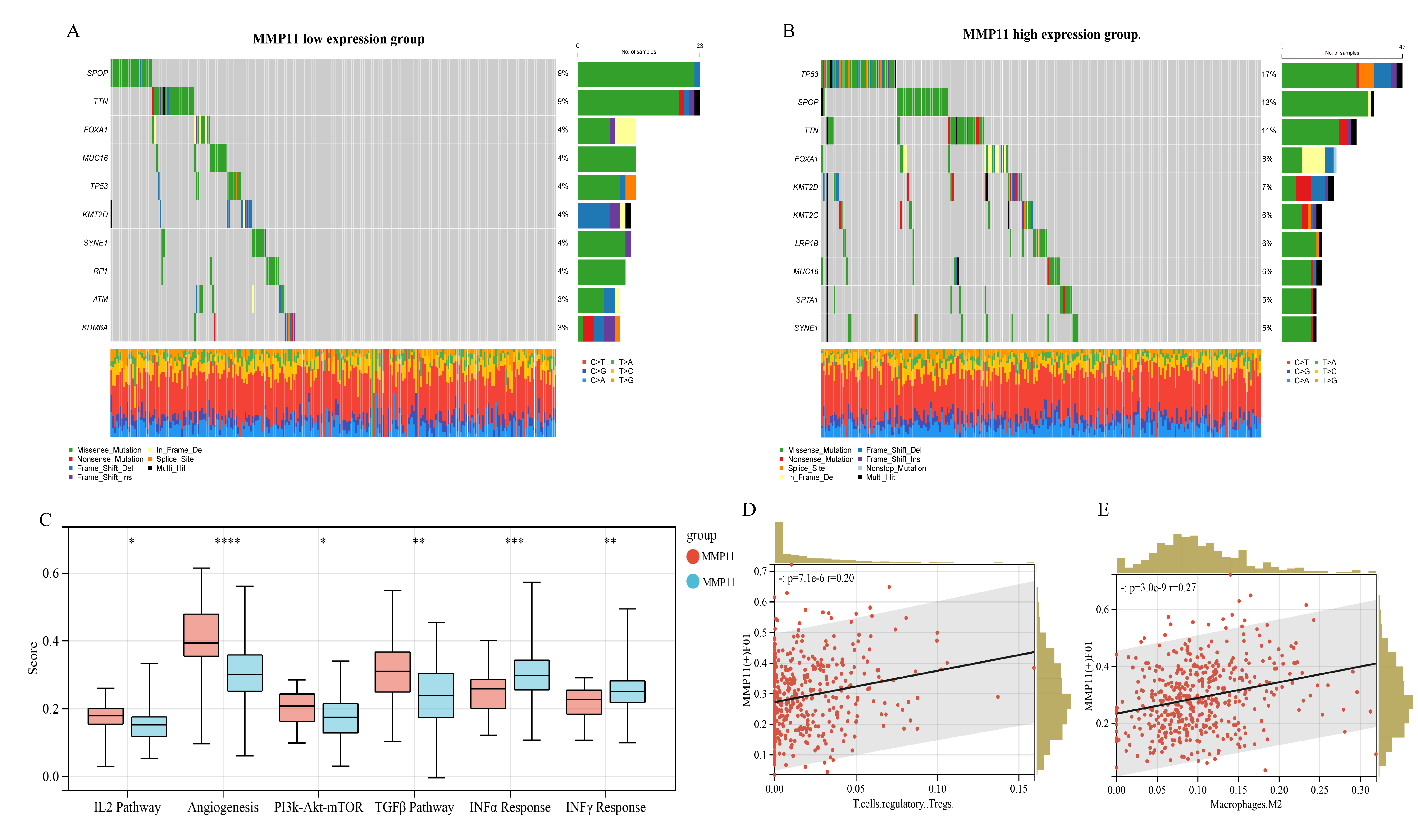


Fig. S3: High risk mutated genes in MMP11 high and low expression groups and characteristics of MMP11 (+) fibroblasts

A: High risk gene mutations in MMP11 low expression group; B: High risk gene mutations in MMP11 high expression group; C: Activation of the MMP11 (+) F01 and MMP11 (-) F01 oncogenic pathways; D: Correlation between MMP11 (+) F01 and Tregs; E: Correlation between MMP11 (+) F01 and macrophages M2;

Note: -＞0.05, *P< 0.05, **P< 0.01, ***P< 0.001, ****P< 0.0001


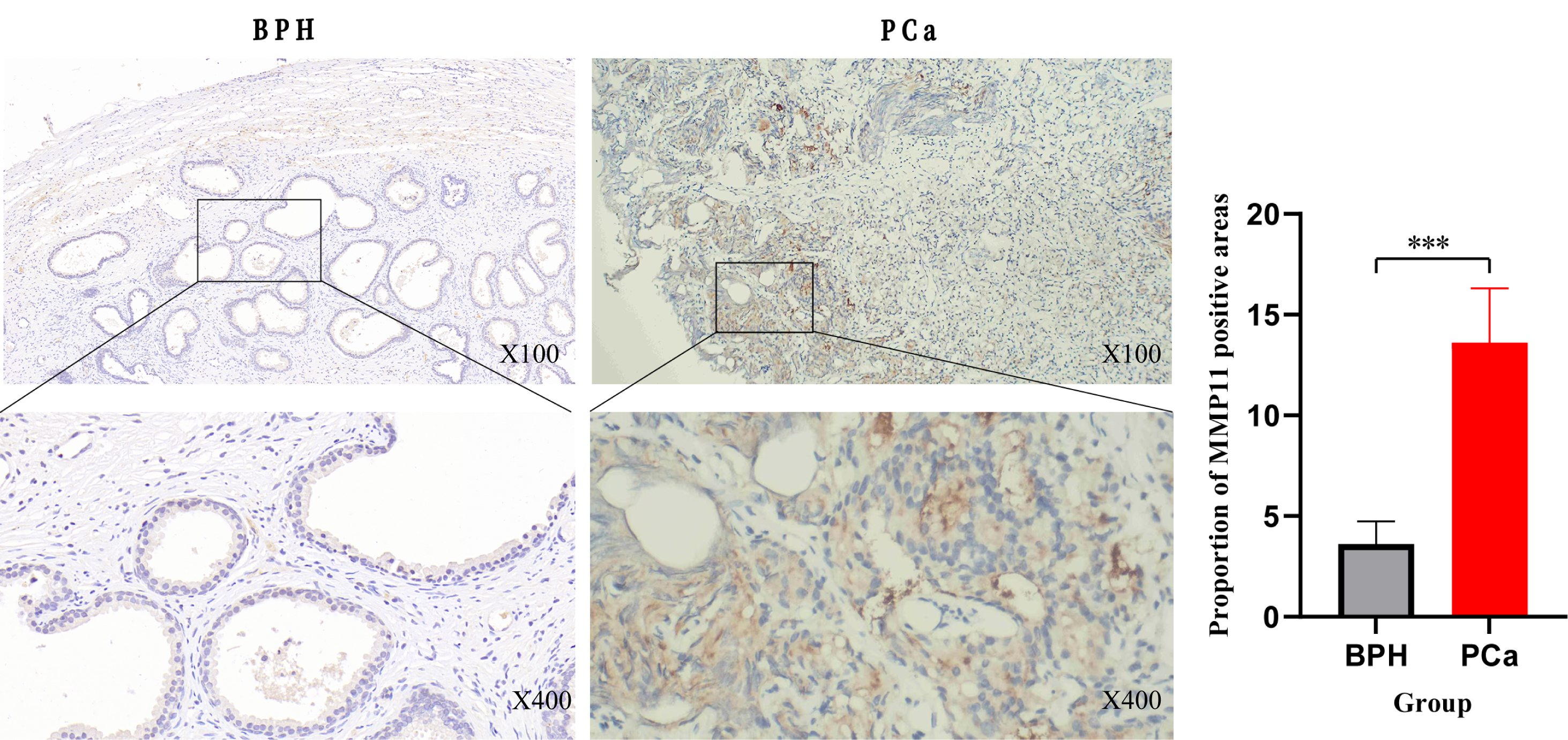


Fig. S4: Immunohistochemical staining intensity of MMP11 in benign prostatic hyperplasia (BPH) and prostate cancer (PCa).

Note: ***P< 0.001


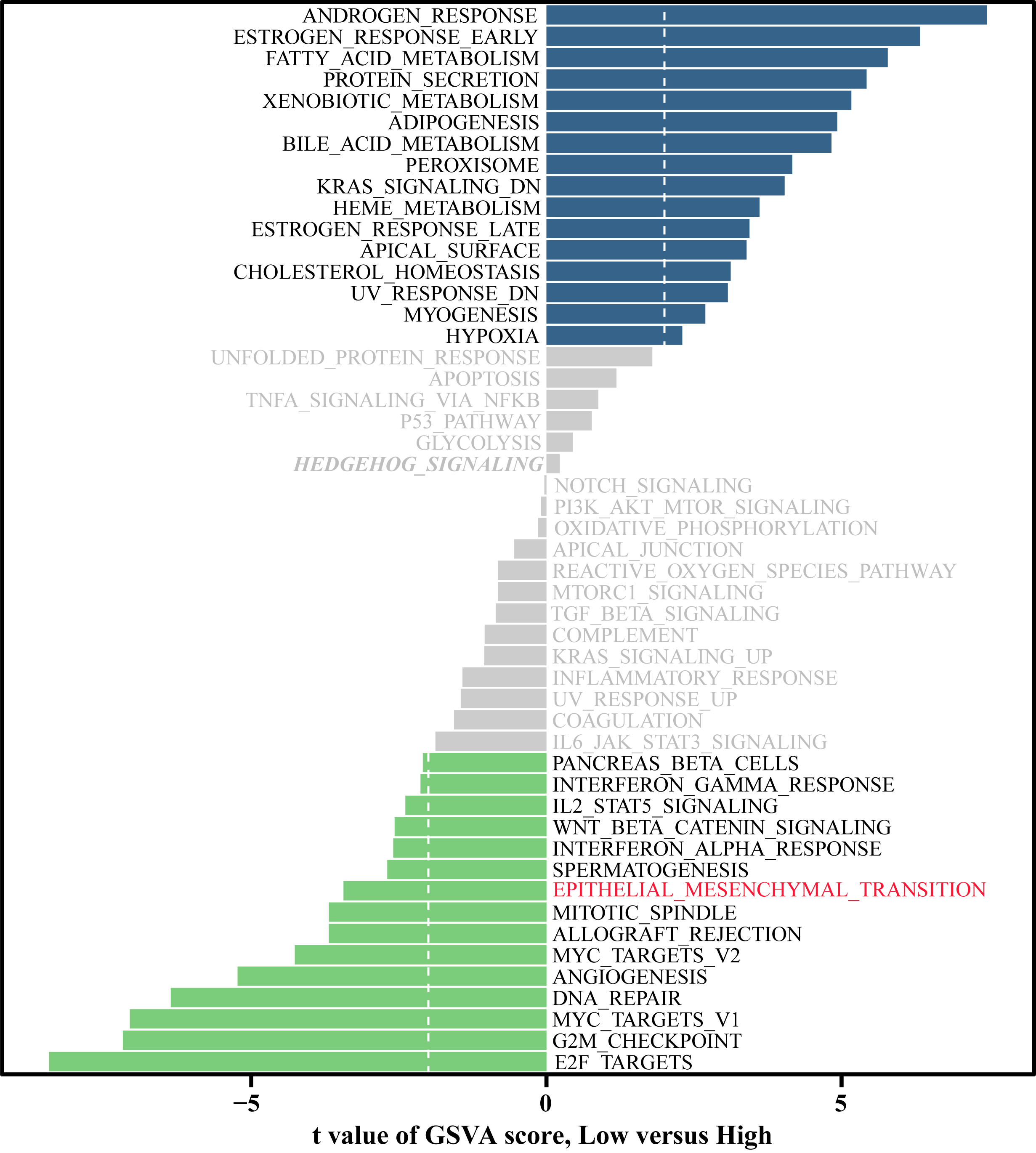


Fig S5: Activation of 50 common oncogenic pathways in MMP11 high and low expression groups


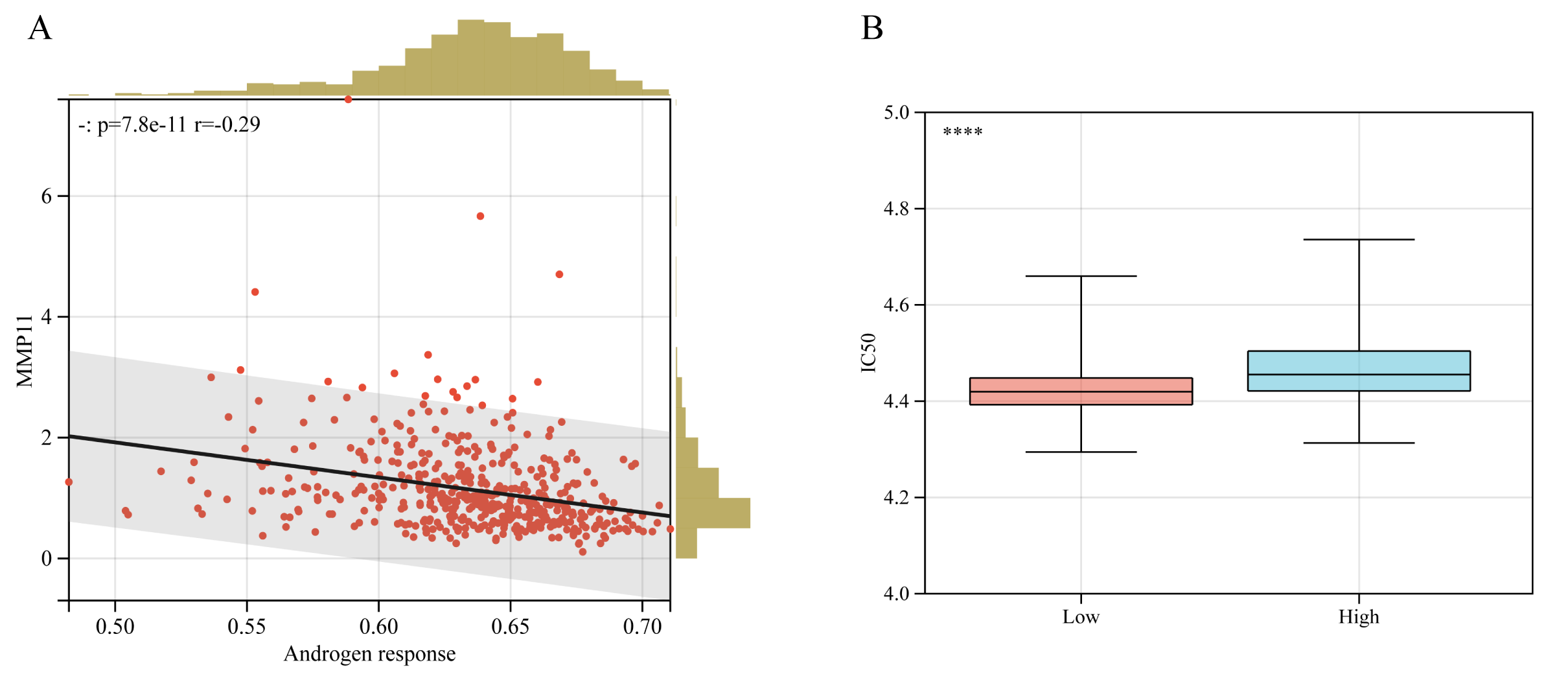


Fig S6: The relationship between MMP11 and androgen receptor (AR) signaling pathway and bicalutamide sensitivity (IC50).
